# Impacts of Compression on the Ground and Low-Lying Excited Doublet States of Plasma-Embedded Lithium Atom

**DOI:** 10.1007/s00601-025-01981-1

**Published:** 2025-02-06

**Authors:** Salah Doma, Gamal Roston, Mostafa Ahmed

**Affiliations:** 1 Department of Mathematics and Computer Science, Alexandria University, Alexandria, 21515 Egypt; 2 Department of Physics, Alexandria University, Alexandria, 21515 Egypt

## Abstract

The variational Monte Carlo method is employed to conduct a comprehensive investigation of the compressed ground and excited states of plasma-embedded lithium atom within impenetrable spherical boxes of varying radii. The study focuses on the low-lying excited doublet states 1$s^{{2}}$*ns*, 1$s^{{2}}n$p, and 1$s^{{2}}n$d, utilizing plasma potentials such as the screened Coulomb (SCP), exponential cosine screened Coulomb (ECSCP), and Hulthén potentials. Energy eigenvalues are determined using appropriate trial wave functions, which account for electron–electron repulsion and spin parts to adhere to the Pauli Exclusion Principle. Moreover, two factors related to the wave function of the compressed system and ECSCP model are considered. The results reveal an intriguing relative ordering for the lithium atom using the three plasma models, with many of the findings being significant contributions yet to be explored.

## Introduction

Grasping the significance of compression impacts on lithium atoms in plasma is essential for advancing nuclear fusion technology. The compression of plasma results in an increase in both the density and temperature of lithium atoms, whereby the atoms (or ions, depending on the ionization state) are forced together in a smaller volume, leading to higher densities as well as higher kinetic energies of these particles, which play a crucial role in facilitating effective fusion reactions. Besides, it not only accelerates reaction rates but also enhances the control of high-energy neutrons generated during fusion. In this context, compressed lithium plays a crucial role in optimizing neutron management, thereby increasing the efficiency and lifespan of the reactor. Consequently, investigating and enhancing the effects of compression on lithium atoms is crucial for developing a sustainable and virtually infinite energy resource for the future.

For dealing with those idealized Coulombic-correlated quantum systems, they are described mainly by the Schrödinger equation and cannot be solved analytically. The problem arises because of the complexity of the involved multidimensional integrals in the calculation of the energy expectation values. Hereby, the choice of an appropriate approximation method must be carefully considered to evaluate the complicated integrals of those many-body systems, as for the lithium atom [1–3]. The Drake variational method [4] was utilized to determine the energy of the lithium atom’s ground state and the excited state $1s^{{2}}3 d$, neglecting the correlation contribution of the wave function, whereas the quantum chemical configuration (CI) method utilized the Cartesian anisotropic Gaussian basis set approach [5] to analyze the electron density distribution and spectrum potential curves of helium and lithium atoms in restricted low-lying states, employing an isotropic harmonic potential for both centered and off-centered positions. In addition, the ground state energy of isoelectronic ions of lithium was studied for the infinite and finite nuclear mass effect [6], while the excitation energies associated with the core states of the lithium atom were measured experimentally [7], whereby the uncertainties in the energy scales for incident and emitted electrons were calculated. Furthermore, the effect of pressure on the ground and the low-lying excited states of few electronic Coulombic system [8], whereas the spectrum of the confined ground and low-lying excited states of the lithium atom was investigated at different radii [9] and isotropic harmonic potentials [10], and the high-lying excited states, using the quantum genetic algorithm procedure and Hartree-Fock Roothaan method for the lithium [11] and beryllium [12] atoms.

Another crucial factor that needs to be considered for a variety of physical systems is the plasma screening effect on atomic processes [13–15], whereby two characteristics of plasmas, particle density n and temperature T, determine whether they are classical or quantum plasmas in the collisional or collisionless regime [16–20]. The exponential-cosine-screened Coulomb potential model [21] was developed to describe dense plasmas in which electrons are regarded as Fermi gas and the electron quantum force resulting from the quantum Bohm potential is considered instead of Fermi pressure. Electron collisions [22] and charge capture processes [23], on the basis of applying the screening effects of this model, have been studied in the plasma environment. In addition, the lithium atom was studied in a weakly coupled plasma environment, where the wave functions were expanded in a correlated Hylleraas-type basis set utilizing the electron–electron correlation effect [24]. Also, the nuclear magnetic shielding constant $(\sigma )$, ionization potential, and diamagnetic susceptibility $(\chi )$ were given in relation to the plasma screening parameter $(\mu )$. Moreover, the lithium atom was studied in both free [25–33] and compressed [34] plasma environments, whereas the low-lying and core excited doublet states, including also the Hulthén potential plasma model [35], were investigated thoroughly at different plasma strengths.

A precise method for approximating complex systems, such as quantum Monte Carlo (QMC) methods, is necessary for solving the Schrödinger equation of many-body quantum systems in various external potentials [36–39]. The main idea behind Monte Carlo numerical techniques is to sample the integrand at random abscissae rather than at every quadrature point. When dealing with high-dimensional integrals, the Monte Carlo method is well-suited for a variety of statistical and quantum mechanical problems. The Monte Carlo quadrature involves two fundamental steps: initially, randomly generating abscissae distributed throughout the integration volume based on a specific distribution *w*(*x*), and subsequently, computing the mean value of the function *f*/*w*, where *f* represents the function being integrated, at these abscissae. An alternative approach must be employed due to the inability to extend techniques for generating random numbers following a specified distribution to effectively sample intricate weight functions in multiple dimensions. The Metropolis algorithm [40] is a widely used method for generating random variables with a specified probability distribution of any shape. The variational Monte Carlo method (VMC), a QMC numerical technique, relies on the variational principle and the Monte Carlo evaluation of integrals using significance sampling based on the Metropolis algorithm. This approach ensures that the statistical error in the integral estimate decreases as the square root of the number of sampled points, regardless of the dimensionality of the problem.

It is important to note that lithium atoms are essential to space science [41] and plasma physics. In order to comprehend and control plasmas in a variety of contexts, including developing and perfecting plasma confinement for potential future fusion energy applications, it serves as a diagnostic tool to measure temperatures and densities in fusion experiments. Furthermore, lithium abundances provide information about the Big Bang nucleosynthesis and aid in our understanding of cosmic-ray interactions and stellar evolution. Accordingly, the purpose of this investigation is to study the impact of compression on the ground and the low-lying excited doublet states of the lithium atom placed at the center of a spherical box under various plasma potentials using the VMC technique.

## Methodology

### The VMC Method

In the variational Monte Carlo (VMC) approach, the calculation of the Hamiltonian operator’s expectation value is achieved through the multiplication and division of the integrand by the trial wave function, as follows

1 $$\begin{aligned} E_{VMC} = \frac{\int {\psi ^{*}(R)\frac{\hat{H}\psi (R)}{\psi (R)} \psi (R)dR} }{\int {\psi ^{*}(R) } \psi (R)dR}, \end{aligned}$$

where $\psi \left( {{{\varvec{R}}}} \right)$ is a trial wave function depending on variational parameters, which are optimized to obtain the minimum energy eigenvalue using importance sampling based on the Metropolis algorithm. We rewrite Eq. (1) as follows:

2 $$\begin{aligned} E_{VMC}= \int {P\left( {\varvec{R}} \right) E_{L}} \left( {\varvec{R}} \right) d({\varvec{R}}), \end{aligned}$$

where $P\left( {\varvec{R}} \right) = \frac{\left| \psi ({\varvec{R}}) \right| ^{2}}{\int \left| \psi ({\varvec{R}}) \right| ^{2} d{\varvec{R}}}$ is interpreted as a probability distribution function and $E_{L}\left( {\varvec{R}} \right) =\frac{\hat{H}\psi \left( {\varvec{R}} \right) }{\psi \left( {\varvec{R}} \right) }$ is the local energy function, which was evaluated using a series of points ${\varvec{R}}_{ij}$ proportional to $P\left( {\varvec{R}} \right)$ according to the Metropolis algorithm. The trial wave function for a given state must produce an energy which is above the exact value of that state; ${E}_{VMC}\ge E_{exact}.$

After enough evaluations, $E_{VMC}$ [42] can be written in the form:

3 $$\begin{aligned} E_{VMC}= <E_{L}>=\mathop {lim}_{N\rightarrow \infty }\mathop {lim}_{M\rightarrow \infty }{\frac{1}{N}\frac{1}{M}}\sum \nolimits _{j=1}^N \sum \limits _{i=1}^M {E_{L}({\varvec{R}}_{ij})}, \end{aligned}$$

where *M* is the ensemble size of generated random points $\left\{ {\varvec{R}}_{1}{,}{\varvec{R}}_{2}{,...,}{\varvec{R}}_{M} \right\}$ and *N* is the number of ensembles. By employing this numerical approach, the acceptance-rejection technique is implemented on random numbers following a probability distribution similar to $\psi ^{2}({\varvec{R}})$ to calculate the local energy. A random number is selected from the probability distribution *P* (***R***) using this method to determine its suitability as a reference. The acceptance criterion is established based on the ratio:

4 $$\begin{aligned} A=\frac{\psi ^{2}\left( {\varvec{R}}_{k} \right) }{\psi ^{2} \left( {\varvec{R}}_{i} \right) }, \end{aligned}$$

which governs the transition probability from an original random number, ${\varvec{R}}_{n}$, to a new random number, ${\varvec{R}}^{{\prime }}.$ If $A > 1$ (i.e., ${\varvec{R}}_{n+1} \quad =$ ${\varvec{R}}^{{\prime }})$, then the new ${\varvec{R}}^{{\prime }}$ becomes a point in the subsequent ensemble, leading to acceptance of the trial step. If $A < 1$, the step is accepted with a probability of *A*. This can be achieved by comparing it with a uniformly distributed random number $(\tau )$ in the range [0, 1] and accepting the step if $\tau < A$. However, if the trial step is rejected, we set ${\varvec{R}}_{n+1} \quad = \quad {\varvec{R}}^{{\prime }}$. This iterative process is carried out for each point in the ensemble to extend later ensembles for broader sampling, with any arbitrary point ${\varvec{R}}_{0}$ serving as the initial point for the random walk. Eventually, it is crucial to consider the energy’s standard deviation when performing computations and providing by

5 $$\begin{aligned} \sigma =\sqrt{\frac{\langle E_L^2 \rangle - \langle {E_L}\rangle ^{2}}{M(N-1)}.} \end{aligned}$$

### The Hamiltonian and Trial Wave Functions

The Hamiltonian operator within the infinite nuclear mass approximation in atomic units (a. u.), $e=\hslash =m=4\pi \varepsilon _{0}=1$, is given by

6 $$\begin{aligned} H=-\frac{1}{2}\mathop {\sum }\limits _{i=1}^3 {({\nabla }_{i}^{2}} +\frac{2Z}{r_{i}} )+\sum \limits _{i<j}^3 {\frac{1}{r_{ij}}}, \end{aligned}$$

where $Z=3$ is the nuclear charge, $r_{i}$ is the distance between the $i^{th}$ electron and the nucleus, and $r_{ij}$ are the inter-electron distances.

Due to the existence of the correlation part, the Hamiltonian using Hylleraas Coordinates [43] is introduced as

7 $$\begin{aligned} H= &   \mathrm {-}\frac{{1}}{{2}}\left( \sum \limits _{i{=1}}^{3} \frac{\partial ^{{2}}}{\partial r_{i}^{{2}}} {+}\sum \limits _{i{=1}}^{3} {\frac{{2}}{r_{i}}\frac{\partial }{\partial r_{i}}} {+}\sum \limits _{i{<}j}^{3} {{2}\frac{\partial ^{{2}}}{\partial r_{ij}^{{2}}}} \mathrm { +}\sum \limits _{i{<}j}^{3} {\frac{\textrm{4}}{r_{ij}}\frac{\partial }{\partial r_{ij}}} \, {+}\sum \limits _{i\mathrm {\ne }j}^{3} \frac{r_{i}^{{2}}{+}r_{ij}^{{2}}\mathrm {-}r_{j}^{{2}}}{r_{i}r_{ij}} \frac{\partial ^{{2}}}{\partial r_{i}\partial r_{ij}}\right. \nonumber \\  &   \left. {+}\sum \limits _{i\mathrm {\ne }j}^{3} \sum \limits _{k\mathrm {>}j}^{3} \frac{r_{ij}^{{2}}{+}r_{ik}^{{2}}\mathrm {-}r_{jk}^{{2}}}{r_{ij}r_{ik}} \frac{\partial ^{{2}}}{\partial r_{ij}\partial r_{ik}}+\sum \limits _{i{=1}}^{3} {\frac{{1}}{r_{i}^{2}}\frac{\partial ^{{2}}}{\partial \theta _{i}^{{2}}}} {+}\sum \limits _{i{=1}}^{3} {\frac{{1}}{r_{i}^{{2}}{sin}^{{2}}\theta _{i}}\frac{\partial ^{{2}}}{\partial \varphi _{i}^{{2}}}{+}} \sum \limits _{i{=1}}^{3} \frac{cot\theta _{i}}{r_{i}^{{2}}}\frac{\partial }{\partial \theta _{i}}\, \right. \nonumber \\  &   \left. {+} \sum \limits _{i\mathrm {\ne }j}^{3} {\left( 2\frac{r_{j}cos\theta _{j}}{r_{i}r_{ij}sin\theta _{i}}\, {+}cot\theta _{i}\frac{{r_{ij}^{{2}}\mathrm {-}r}_{i}^{{2}}\mathrm {-}r_{j}^{{2}}}{r_{i}^{{2}}r_{ij}} \right) \frac{\partial ^{{2}}}{\partial \theta _{i}\partial r_{ij}}} {+}+\sum \limits _{i\mathrm {\ne }j}^{3} {{2}\frac{r_{j}sin\theta _{j}}{r_{i}r_{ij}sin\theta _{i}}\sin \left( \varphi _{i}\mathrm {-}\varphi _{j} \right) \frac{\partial ^{{2}}}{\partial \varphi _{i}\partial r_{ij}}} \right) \nonumber \\  &   +\sum \limits _{i{=1}}^{3} \frac{\mathrm {-}Z}{r_{i}} {+}\sum \limits _{i{<}j}^{3} \frac{{1}}{r_{ij} } {.} \end{aligned}$$

For plasma models, in hot dense plasma, the collective impacts of correlated many-particle interactions give rise to screened Coulomb interactions, which are represented by the SCP and given by

8 $$\begin{aligned} V_{SCP}\left( r \right) =-\frac{Ze^{2}}{r}exp(-\mu r), \end{aligned}$$

where $\mu =\frac{1}{\lambda _{D}}$ represents the Debye screening parameter and it determines the interaction between electron–electron in Debye plasma. It depends on the temperature and the density of the plasma in the following form [44]

9 $$\begin{aligned} \mu =\frac{1}{\lambda _{D}}=\sqrt{4\pi e^{2}N_{e}/KT_{e}}, \end{aligned}$$

where $\lambda _{D}$ is called the Debye screening length, *K* is the Boltzmann constant, $T_{e}$ is the electron temperature, *e* is the electronic charge, and $N_{e}$ is the plasma-electron density.

The study of effective screened potential in dense quantum plasmas or the ECSCP [45] is introduced as

10 $$\begin{aligned} V_{ECSCP}\left( r \right) =-\frac{Ze^{2}}{r}exp\left( -\mu r \right) \cos \left( \mu r \right) . \end{aligned}$$

Furthermore, the Hulthén potential [46] is given by

11 $$\begin{aligned} V_{Hu}\left( r \right) =-Ze^{2}\frac{\mu e^{-\mu r}}{1-e^{-\mu r}}. \end{aligned}$$

Accordingly, the Hamiltonian by considering the SCP is given by:

12 $$\begin{aligned} H_{1}=-\frac{1}{2}\sum \limits _{i=1}^3 {\nabla }_{i}^{2} -3\sum \limits _{i{=1}}^{3} \frac{\exp \left( -\mu r_{i} \right) }{r_{i}} {+}\sum \limits _{i{<}j}^{3} {\frac{\exp \left( -\mu r_{ij} \right) }{r_{ij} }, } \end{aligned}$$

and with the ECSCP is given by:

13 $$\begin{aligned} H_{2}=-\frac{1}{2}\sum \limits _{i=1}^3 {\nabla }_{i}^{2} -3\sum \limits _{i{=1}}^{3} \frac{\exp {\left( -\mu r_{i} \right) \textrm{cos}(\mu r_{i})}}{r_{i}} {+}\sum \limits _{i{<}j}^{3} {\frac{\exp {\left( -\mu r_{ij} \right) \cos \left( \mu r_{ij} \right) }}{r_{ij} }. } \end{aligned}$$

But for the Hulthén potential model, it is given by

14 $$\begin{aligned} H_{3}=-\frac{1}{2}\sum \limits _{i=1}^3 {\nabla }_{i}^{2} -3\sum \limits _{i{=1}}^{3} \frac{\mu e^{-\mu r_{i}}}{1-e^{-\mu r_{i}}} {+}\sum \limits _{i{<}j}^{3} {\frac{\mu e^{-\mu r_{ij}}}{1-e^{-\mu r_{ij}}}.} \end{aligned}$$

Here, the kinetic term in Eqs. (12), (13), and (14) is introduced in the form of Hylleraas coordinates, as in Eq. (7).

In case of the confined system by an impenetrable spherical box, the total Hamiltonian is

15 $$\begin{aligned} H_{Conf}=H_{p}+V_{C} \left( r_{1},r_{2},r_{3} \right) , \end{aligned}$$

where $H_{p} = (H_{1},H_{2},H_{3})$ is the Hamiltonian of the used plasma model and $V_{C}\left( r_{{1}}{,}r_{{2}}{,}r_{{3}} \right)$ is the confined potential due to the box of radius $R_{C}$, and is given by

16 $$\begin{aligned} V_{C}\left( r_{{1}}{,}r_{{2}}{,}r_{\textrm{3}} \right) {=}\left\{ {\begin{array}{cc} {0,}&  r_{{1}}{,}\,r_{{2}}{,}\, r_{\textrm{3}}\,{<}\, Rc\\ {\infty ,}&  r_{{1}}{,}\,r_{{2}}{,}\,r_{\textrm{3}},\,{\ge }\,Rc \\ \end{array}} \right. . \end{aligned}$$

The trial wave functions employed for the ground and low-lying excited doublet states of the lithium atom within the SCP, ECSCP, and Hulthén models are presented as

17 $$\begin{aligned} \varPsi _{SCP\,, Hu}\left( {\varvec{r}}_{{1}}{,}{\varvec{r}}_{{2}}{,}{\varvec{r}}_{\textrm{3}} \right) {=}\mathcal {A}\left[ \psi \left( {\varvec{r}}_{{\textbf{1}}}{,}{\varvec{r}}_{{\textbf{2}}}{,}{\varvec{r}}_{{\textbf{3}}} \right) \chi (1,2,3)\prod \limits _{i{<}j} {f\left( r_{ij} \right) } \right] \prod \limits _i \left( 1-\frac{r_{i}}{Rc} \right) , \end{aligned}$$

and

18 $$\begin{aligned} \varPsi _{ECSCP }\left( {\varvec{r}}_{{1}}{,}{\varvec{r}}_{{2}}{,}{\varvec{r}}_{\textrm{3}} \right) {=}\mathcal {A}\left[ \psi \left( {\varvec{r}}_{{\textbf{1}}}{,}{\varvec{r}}_{{\textbf{2}}}{,}{\varvec{r}}_{{\textbf{3}}} \right) \,\chi \,(1,2,3)\prod \limits _{i{<}j} {f\left( r_{ij} \right) } \right] \prod \limits _i \left( \mathrm {1-}\mu r_{i} \right) \,\prod \limits _i \left( 1-\frac{r_{i}}{Rc} \right) , \end{aligned}$$

where $\mathcal {A}$ is the antisymmetrizer [47]

19 $$\begin{aligned} \mathcal {A}\quad = \quad I\mathrm {-}\hat{P}_{12} \quad - \quad \hat{P}_{23} \quad - \quad \hat{P}_{31} +\hat{P}_{123} \quad +\hat{P}_{132}. \end{aligned}$$

Here *I* is the identity permutation, while $\hat{P}_{ij\,}$ is the permutation of the $i^{th}$ and $j^{th}$ particles. Analogously, the operator $\hat{P}_{ijk\,}$ is the permutation of three electrons *i*, *j*, and *k*. The wave function $\psi \,\left( {\varvec{r}}_{{\textbf{1}}}, {\varvec{r}}_{{\textbf{2}}},{\varvec{r}}_{{\textbf{3}}} \right)$ represents the spatial distribution of a hydrogen-like atom in the *nl* state, characterized also by radial wave functions corresponding to the 4*l* states [48–50]. This can be expressed as:

20 $$\begin{aligned} \psi \left( {\varvec{r}}_{{\textbf{1}}},{\varvec{r}}_{{\textbf{2}}},{\varvec{r}}_{{\textbf{3}}} \right) \quad = \quad \psi _{Z^{'}}\left( r_{1} \right) {\,\psi }_{Z^{'}}\left( r_{2} \right) {\,R}_{nl}(Z^{''},r_{3})\,Y_{lm}(\theta _{3},\varphi _{3}), \end{aligned}$$

and the spin function for $1 s^{2}$*ns* and $1s^{2}$ *nd* states, which must be anti-symmetric to satisfy properly the Pauli exclusion principle, is given by

21a $$\begin{aligned} \chi \,\left( 1,2,3 \right) =\,\alpha \left( {1} \right) \beta \left( {2} \right) \beta \left( {3} \right) -\beta ({1})\,\alpha ({2})\beta (\mathrm {3)}. \end{aligned}$$

But for $1 s^{2}\, {np}$ states, they have mixed symmetry because they are neither fully symmetric nor anti-symmetric under the exchange of any two electrons, and hence the spin function after applying the antisymmetrizer $\mathcal {A}$ is introduced as

21b $$\begin{aligned} \chi \,\left( 1,2,3 \right) =\left[ \left( \beta \left( {1} \right) \beta \left( {2} \right) \alpha \left( {3} \right) -\beta \left( {1} \right) \alpha \left( {2} \right) \beta \left( {3} \right) \right) \,+\left( \beta \left( {1} \right) \beta \left( {2} \right) \alpha \left( {3} \right) \mathrm {-}\alpha \left( {1} \right) \beta \left( {2} \right) \beta \left( {3} \right) \right) \right] , \end{aligned}$$

where $\alpha$ and $\beta$ are the spinor indices. Also, the Jastrow correlation function $f\left( r_{ij} \right)$ [51] is given by

22 $$\begin{aligned} f\left( r_{ij} \right) \mathrm {=exp}\left[ \frac{r_{ij}}{\varepsilon \,\left( \mathrm {1+}\sigma \,r_{ij} \right) } \right] . \end{aligned}$$

Here, the variational parameters $z^{'},\,z^{''},\,$ and $\sigma$ are finely varied in order to obtain the best fit to the energy eigenvalues, while $\varepsilon \,\,$ makes $f\left( r_{ij} \right)$ satisfy the cusp conditions, as $$\varepsilon =\left\{ {\begin{array}{l} \,2\,\,\,for\,unlike\,spins\,\\ 4\,\,\,\,\,\,\,\,for\,like\,spins \\ \end{array}} \right. $$. Also, $\prod \limits _i \left( \mathrm {1-}\mu r_{i} \right)$ is the effective-plasma factor related to the ECSCP model only provided that $r_{i}{<\,}\frac{1}{\mu }$ and $\varPsi _{ECSCP}\mathrm {=0}$ at $r_{i}{=\,}\frac{1}{\mu }$, whereas the cut-off factor $\prod \limits _i \left( \mathrm {1-}\frac{r_{i}}{Rc} \right)$ satisfies Dirichlet boundary conditions at which $\varPsi _{SCP,\,\,Hu}{=}\varPsi _{ECSCP}\mathrm {=0}$ at $r_{i}\mathrm {=\,}Rc$.

### The Qualitative Aspects of the Model Potentials

For the compressed atom inside an impermeable spherical box, its internal energy is increased, possibly leading to higher kinetic energy for the entire system. Also, the atom would continue to exert pressure on the hard walls of the box due to its internal state, as the hard walls exert an instantaneous infinite force at the boundary, while for the soft walls (penetrable walls) of definite potentials the atoms or particles experiences gradually increases forces as they approach the edges of the confinement. In addition, the energy level may shift accordingly, which leads to changes in the wave-like nature of the atom. Hereby, it can affect the spatial extent of the wave function, potentially altering the probability of finding the atom in different regions of the box.

Conversely, an atom can be found in either the collisional or collisionless domain when influenced by plasmas, depending on densities and temperatures. This results in two plasma regimes: classical or quantum plasmas. Weakly connected plasmas, known as the classical regime, are characterized by the predominance of thermal motion, making collective or collisionless effects more noticeable. The potential of an atom in such plasmas is described by the screened Coulomb potential (SCP). On the other hand, the strongly coupled (collisional) plasma, known as the quantum regime, exhibits more prominent quantum effects due to the substantial overlap of bound state wave functions with neighboring plasma particles. In this regime, there is an increase in plasma density at low temperatures, and the potential energy becomes more significant than the kinetic energy. The electrons in this regime are considered Fermi gas, and the electron quantum force resulting from the quantum Bohm potential is involved, while the quantum statistical Fermi-pressure is disregarded. Therefore, dense quantum plasmas are proposed to be characterized by the exponential-cosine-screened Coulomb potential (ECSCP). Consequently, we utilized the ECSCP model as a confined system to study the behavior of the lithium atom when impacted by plasma particles. In contrast to the compression impact, the confined ECSCP model demonstrates a clear effective plasma potential and a rapid decrease in the correlation part. On the other hand, the compression impact always maintains a permanent zero confined potential within the box, along with an increase in the correlation part (electron–electron repulsion) as the box radii decrease.

In addition to such plasma models, it’s also worth mentioning that the Hulthén potential is used in plasma physics and quantum mechanics to model interactions between particles in plasma or a solid-state environment. It can alter the binding energy of an atom in plasma, which has an attractive component that can bind electrons more strongly to the nucleus compared to a simpler Coulomb potential, depending on the parameters of the potential. Also, it has an impact on the wave functions of electrons within the atom, whereby the shape of the wave function can be distorted and change the probability distribution of electrons at a given position. Furthermore, the energy levels of the atom can be shifted, which can affect the atom’s spectral lines and its overall energy states. The Hulthén potential is characterized mathematically by a limited range, displaying Coulomb-like characteristics for small *r* and exponentially decreasing behavior for larger *r*. Furthermore, as $\mu$ tends towards zero, it transforms into a Coulomb potential, and as indicated in Eq. (11), it models a three-dimensional delta function $\delta (r)$ as $\mu$ approaches infinity.

## Results and Discussion

In this study, the VMC numerical technique was employed, employing $10^{\textrm{7}}$ Monte Carlo integration points, to calculate the minimum energy eigenvalues with reduced statistical uncertainties for the ground and low-lying excited doublet states of the lithium atom, along with a standard deviation on the order of $10^{\mathrm {-5}}$. Our computational software allows us to systematically adjust the variational parameters through iterations, where the computational code for each system was developed by incorporating the Metropolis algorithm in the variational Monte Carlo approach. Our work employs two different mechanisms to characterize the electronic structure and energy states of the lithium atom under the influence of external potentials with different strengths.

**Table 1:** Energies of ground and low-lying excited doublet states of lithium atom using the SCP, ECSCP, and Hulthén models at various values of confinement radii (*Rc*), in a.u

*Rc*	$\mu$	1$s^{{2}}$2*s*	1$s^{{2}}$2*p*	1$s^{{2}}$3*s*	1$s^{{2}}$3*p*	1$s^{{2}}$3*d*	1$s^{{2}}$4*s*	1$s^{{2}}$4*p*	1$s^{{2}}$4*d*
*The SCP model*									
1	0	9.91804	2.829000	20.63940	13.87180	9.04850	49.52130	32.42330	23.176600
	0.05	11.7038	4.58378	22.3941	15.6265	10.8032	51.27608	34.17808	24.931384
	0.1	13.4226	6.25488	24.0652	17.2976	12.4743	52.94719	35.84919	26.602486
	0.5	23.9968	16.838748	34.6491	27.8815	23.0582	63.53105	46.43305	37.186348
	1	31.8314	24.673368	42.4837	35.7161	30.8928	71.36567	54.26767	45.020968
2	0	-4.44996	-5.668000	-2.70150	-3.274800	-4.03740	5.15570	-0.16590	-0.773600
	0.05	-2.9618	-4.205680	-1.2391	-1.812480	-2.5750	6.61802	1.29642	0.688720
	0.1	-1.5294	-2.813095	0.15340	-0.419895	-1.1825	8.01061	2.68901	2.081305
	0.5	7.28240	6.00679	8.97329	8.39999	7.63739	16.83049	11.50889	10.901190
	1	13.8112	12.535640	15.5021	14.928840	14.1662	23.35934	18.03774	17.430040
3	0	-6.48036	-6.809000	-5.83520	-5.849100	-5.98880	-2.38730	-3.88340	-4.758200
	0.05	-5.2898	-5.639144	-4.6653	-4.679244	-4.8189	-1.21744	-2.71354	-3.588344
	0.1	-4.1439	-4.525076	-3.5512	-3.565176	-3.7048	-0.10338	-1.59948	-2.474276
	0.5	2.90553	2.53083	3.50463	3.49073	3.35103	6.95253	5.45643	4.581632
	1	8.12861	7.75391	8.72771	8.71381	8.57411	12.17561	10.67951	9.804712
5	0	-7.28386	-7.276500	-7.14950	-7.181900	-6.91800	-6.16960	-6.53800	-6.565500
	0.05	-6.3909	-6.399108	-6.2721	-6.304508	-6.0406	-5.29221	-5.66061	-5.688108
	0.1	-5.5315	-5.563557	-5.4365	-5.468957	-5.2050	-4.45666	-4.82506	-4.852557
	0.5	-0.2444	-0.271626	-0.1446	-0.177026	0.08687	0.83527	0.46687	0.439374
	1	3.67287	3.64568	3.77268	3.74028	4.00418	4.75258	4.38418	4.356684
8	0	-7.45076	-7.386500	-7.33090	-7.322000	-7.19820	-7.19390	-7.20260	-7.138600
	0.05	-6.8555	-6.801572	-6.7459	-6.737072	-6.6132	-6.60897	-6.61767	-6.553672
	0.1	-6.2825	-6.244538	-6.1889	-6.180038	-6.0562	-6.05194	-6.06064	-5.996638
	0.5	-2.7578	-2.716584	-2.6609	-2.652084	-2.5282	-2.52398	-2.53268	-2.468684
	1	-0.1462	-0.105044	-0.0494	-0.040544	0.08326	0.08756	0.07886	0.142856
10	0	-7.46966	-7.401600	-7.34550	-7.328600	-7.23690	-7.309700	-7.30330	-7.302600
	0.05	-7.1720	-7.109136	-7.0530	-7.036136	-6.9444	-7.017236	-7.01084	-7.010136
	0.1	-6.8855	-6.830619	-6.7745	-6.757619	-6.6659	-6.738719	-6.73232	-6.731619
	0.5	-5.1231	-5.066642	-5.0105	-4.993642	-4.9019	-4.974742	-4.96834	-4.967642
	1	-3.8174	-3.760872	-3.7047	-3.687872	-3.5961	-3.668972	-3.66257	-3.661872
$\infty$	0	-7.4780	-7.410154	-7.354073	-7.337198	-7.335522	-7.318347	-7.311926	-7.311193
	0.05	-7.180393	-7.117690	-7.061609	-7.044734	-7.043058	-7.025883	-7.019462	-7.018729
		-7.18555[24]		-7.09698[24]					
	0.1	-6.893926	-6.839173	-6.783092	-6.766217	-6.764541	-6.747366	-6.740945	-6.740212
		-6.90707[24]							
	0.5	-5.131548	-5.075196	-5.019115	-5.002240	-5.000564	-4.983389	-4.976968	-4.976235
	1	-3.825778	-3.769426	-3.713345	-3.696470	-3.694794	-3.677619	-3.671198	-3.670465
*The ECSCP model*									
1	0.05	13.12426	6.708924	24.51932	17.75169	12.92841	53.40118	36.30318	27.056518
	0.1	17.93216	8.487564	26.29796	19.53039	14.70705	55.17988	38.08188	28.835158
	0.5	26.94957	19.860744	37.67114	30.90357	26.08023	66.55306	49.45506	40.208338
	1	31.99001	27.661164	45.17156	38.34399	33.52071	73.99348	56.89548	47.648758
2	0.05	-1.77811	-2.434730	0.53176	-0.041560	-0.80414	8.38894	3.06733	2.459665
	0.1	2.22848	-0.952530	2.01397	1.440690	0.67806	9.87119	4.54958	3.941865
	0.5	9.74298	8.525120	11.49162	10.918340	10.15571	19.34884	14.02723	13.419515
	1	13.94335	15.025470	17.74197	17.118690	16.35611	25.54919	20.22758	19.619865
3	0.05	-4.34288	-4.222384	-3.24859	-3.262508	-3.40219	0.19929	-1.29682	-2.171588
	0.1	-1.13761	-3.036624	-2.06283	-2.076708	-2.21643	1.38509	-0.11102	-0.985828
	0.5	4.87399	4.545496	5.51929	5.505412	5.36569	8.96721	7.47110	6.596292
	1	8.23428	9.745776	10.51957	10.465692	10.32601	13.92749	12.43138	11.556572
5	0.05	-5.68075	-5.336538	-5.20954	-5.241956	-4.97804	-4.22966	-4.59806	-4.625541
	0.1	-3.27680	-4.447218	-4.32022	-4.352606	-4.08872	-3.34031	-3.70871	-3.736221
	0.5	1.23190	1.239372	1.36637	1.333984	1.59787	2.34628	1.97788	1.950369
	1	3.75212	5.139582	5.11658	5.054194	5.31811	6.06649	5.69809	5.670579

*Rc*	$\mu$	1$s^{{2}}$2*s*	1$s^{{2}}$2*p*	1$s^{{2}}$3*s*	1$s^{{2}}$3*p*	1$s^{{2}}$3*d*	1$s^{{2}}$4*s*	1$s^{{2}}$4*p*	1$s^{{2}}$4*d*
8	0.05	-6.38202	-6.093192	-6.03759	-6.028704	-5.90490	-5.90061	-5.90931	-5.845294
	0.1	-4.77939	-5.500312	-5.44471	-5.435804	-5.31202	-5.30771	-5.31641	-5.252414
	0.5	-1.77358	-1.709252	-1.65365	-1.644744	-1.52096	-1.51665	-1.52535	-1.461354
	1	-0.09344	0.890888	0.84649	0.835396	0.95920	0.96349	0.95479	1.018786
10	0.05	-6.93529	-6.754946	-6.69885	-6.681952	-6.59025	-6.663053	-6.65665	-6.655947
	0.1	-6.13397	-6.458506	-6.40241	-6.385502	-6.29381	-6.366603	-6.36020	-6.359507
	0.5	-4.63107	-4.562976	-4.50688	-4.489972	-4.39828	-4.471073	-4.46467	-4.463977
	1	-3.79100	-3.262906	-3.25681	-3.249902	-3.15820	-3.231003	-3.22460	-3.223907
$\infty$	0	-7.47802	-7.41015	-7.35407	-7.3372	-7.33552	-7.31835	-7.31193	-7.31119
	0.05	-6.534960	-6.76350	-6.70742	-6.69055	-6.68887	-6.67170	-6.66528	-6.66454
	0.1	-6.142333	-6.46706	-6.41098	-6.39410	-6.39243	-6.37525	-6.36883	-6.36810
	0.5	-4.515450	-4.57153	-4.51545	-4.49857	-4.49690	-4.47972	-4.47330	-4.47257
	1	-3.265380	-3.27146	-3.26538	-3.25850	-3.25682	-3.23965	-3.23323	-3.23250
*The Hulthén model*									
1	0.05	11.24330	4.154256	21.96466	15.19706	10.37376	50.84656	33.74856	24.501856
	0.1	12.50565	5.416614	23.22701	16.45941	11.63611	52.10891	35.01091	25.764214
	0.5	20.65895	13.569906	31.38031	24.61271	19.78941	60.26221	43.16421	33.917506
	1	27.11765	20.028612	37.83901	31.07141	26.24811	66.72091	49.62291	40.376212
2	0.05	-3.34558	-4.563620	-1.59712	-2.170420	-2.93302	6.26008	0.93848	0.330780
	0.1	-2.29362	-3.511655	-0.54516	-1.118455	-1.88106	7.31205	1.99045	1.382745
	0.5	4.50080	3.282755	6.24926	5.675955	4.91336	14.10646	8.78486	8.177155
	1	9.88305	8.665010	11.63151	11.058210	10.29561	19.48871	14.16711	13.559410
3	0.05	-5.59686	-5.925496	-4.95170	-4.965596	-5.10530	-1.50380	-2.99990	-3.874696
	0.1	-4.75528	-5.083924	-4.11012	-4.124024	-4.26372	-0.66222	-2.15832	-3.033124
	0.5	0.68024	0.351604	1.32540	1.311504	1.17180	4.77330	3.27720	2.402404
	1	4.98605	4.657408	5.63121	5.617308	5.47761	9.07911	7.58301	6.708208
5	0.05	-6.62123	-6.613872	-6.48687	-6.519272	-6.25537	-5.50697	-5.87537	-5.902872
	0.1	-5.99005	-5.982693	-5.85569	-5.888093	-5.62419	-4.87579	-5.24419	-5.271693
	0.5	-1.91341	-1.906047	-1.77905	-1.811447	-1.54755	-0.79915	-1.16755	-1.195047
	1	1.31595	1.323306	1.45031	1.417906	1.68181	2.43021	2.06181	2.034306
8	0.05	-7.00901	-6.944748	-6.88915	-6.880248	-6.75645	-6.75215	-6.76085	-6.696848
	0.1	-6.58822	-6.523962	-6.46836	-6.459462	-6.33566	-6.33136	-6.34006	-6.276062
	0.5	-3.87046	-3.806198	-3.75060	-3.741698	-3.61790	-3.61360	-3.62230	-3.558298
	1	-1.71756	-1.653296	-1.59770	-1.588796	-1.46500	-1.46070	-1.46940	-1.405396
10	0.05	-7.24878	-7.180724	-7.12462	-7.107724	-7.01602	-7.088824	-7.08242	-7.081724
	0.1	-7.03839	-6.970331	-6.91423	-6.897331	-6.80563	-6.878431	-6.87203	-6.871331
	0.5	-5.67951	-5.611449	-5.55535	-5.538449	-5.44675	-5.519549	-5.51315	-5.512449
	1	-4.60306	-4.534998	-4.47890	-4.461998	-4.37030	-4.443098	-4.43670	-4.435998
$\infty$	0	-7.47802	-7.410154	-7.354073	-7.337198	-7.335522	-7.318347	-7.311926	-7.311193
	0.05	-7.25714	-7.189278	-7.133197	-7.116322	-7.114646	-7.097471	-7.091050	-7.090317
	0.1	-7.04675	-6.978885	-6.922804	-6.905929	-6.904253	-6.887078	-6.880657	-6.879924
	0.5	-5.68787	-5.620003	-5.563922	-5.547047	-5.545371	-5.528196	-5.521775	-5.521042
	1	-4.61142	-4.543552	-4.487471	-4.470596	-4.468920	-4.451745	-4.445324	-4.444591

**Table 2:** Energies of confined ground and low-lying excited doublet states of lithium atom at various values of *Rc* compared to previous findings, in a.u

*Rc*	1$s^{{2}}$2*s*	1$s^{{2}}$2*p*	1$s^{{2}}$3*s*	1$s^{{2}}$3*p*	1$s^{{2}}$3*d*	1$s^{{2}}$4*s*	1$s^{{2}}$4*p*	1$s^{{2}}$4*d*
1	9.91804	2.82900	20.63940	13.87180	9.04850	49.52130	32.42330	23.17660
	8.29080$^{\mathrm {\,a}}$	1.6104$^{\mathrm {\,a\,\,}}$	20.5775$^{\mathrm {\,a}}$	13.8344$^{\mathrm {\,a}}$	9.0559$^{\mathrm {\,a}}$	49.6270$^{\mathrm {\,a}}$	32.5020$^{\mathrm {\,a}}$	23.3630$^{\mathrm {\,a}}$
	6.518252$^{\mathrm {\,b}}$	0.761377$^{\mathrm {\,b}}$						
2	-4.44996	-5.66800	-2.70150	-3.27480	-4.03740	5.15570	-0.16590	-0.77360
	-5.17540$^{\mathrm {\,a}}$	-6.0223$^{\mathrm {\,a}}$	-2.7634$^{\mathrm {\,a}}$	-3.3122$^{\mathrm {\,a}}$	-4.0300$^{\mathrm {\,a}}$	5.2614$^{\mathrm {\,a}}$	-0.0872$^{\mathrm {\,a}}$	-0.5872$^{\mathrm {\,a}}$
	-5.308362$^{\mathrm {\,b}}$	-5.923905$^{\mathrm {\,b}}$						
3	-6.48036	-6.80900	-5.83520	-5.84910	-5.98880	-2.38730	-3.88340	-4.75820
	-6.8013$^{\mathrm {\,a}}$	-6.9333$^{\mathrm {\,a}}$	-5.8971$^{\mathrm {\,a}}$	-5.8865$^{\mathrm {\,a}}$	-5.9814$^{\mathrm {\,a}}$	-2.2816$^{\mathrm {\,a}}$	-3.8047$^{\mathrm {\,a}}$	-4.5718$^{\mathrm {\,a}}$
	-6.866611$^{\mathrm {\,b}}$	-6.883708$^{\mathrm {\,b}}$						
5	-7.28386	-7.27650	-7.14950	-7.181900	-6.91800	-6.16960	-6.53800	-6.56550
	-7.3386$^{\mathrm {\,a}}$	-7.2524$^{\mathrm {\,a}}$	-7.2114$^{\mathrm {\,a}}$	-7.2193$^{\mathrm {\,a}}$	-6.9106$^{\mathrm {\,a}}$	-6.0639$^{\mathrm {\,a}}$	-6.4593$^{\mathrm {\,a}}$	-6.3791$^{\mathrm {\,a}}$
	-7.387625$^{\mathrm {\,b}}$	-7.293848$^{\mathrm {\,b}}$						
8	-7.45076	-7.38650	-7.33090	-7.32200	-7.19820	-7.19390	-7.20260	-7.13860
	-7.4061$^{\mathrm {\,a}}$	-7.3545$^{\mathrm {\,a}}$	-7.3928$^{\mathrm {\,a}}$	-7.3594$^{\mathrm {\,a}}$	-7.1908$^{\mathrm {\,a}}$	-7.0882$^{\mathrm {\,a}}$	-7.1239$^{\mathrm {\,a}}$	-6.9522$^{\mathrm {\,a}}$
	-7.470666$^{\mathrm {\,b}}$	-7.392324$^{\mathrm {\,b}}$						
10	-7.46966	-7.40160	-7.34550	-7.32860	-7.23690	-7.30970	-7.30330	-7.30260
	-7.4175$^{\textrm{a}}$	-7.3606$^{\mathrm {\,a}}$	-7.4074$^{\mathrm {\,a}}$	-7.3660$^{\mathrm {\,a}}$	-7.2295$^{\mathrm {\,a}}$	-7.2040$^{\mathrm {\,a}}$	-7.2246$^{\mathrm {\,a}}$	-7.1162$^{\mathrm {\,a}}$
	-7.476757$^{\mathrm {\,b}}$	-7.404783$^{\mathrm {\,b}}$						
$\infty$	-7.47802	-7.410154	-7.354073	-7.337198	-7.335522	-7.318347	-7.311926	-7.311193
	-7.47806$^{\textrm{c}}$	-7.410156 $^{\textrm{c}}$	-7.354098$^{\textrm{c}}$		-7.335523$^{\textrm{c}}$	-7.31853$^{\textrm{c}}$		
	-7.478054$^{\textrm{b}}$	-7.409845$^{\mathrm {\,b\,}}$						
	-7.47798$^{\textrm{d}}$							
	-7.48082$^{\textrm{e}}$							

$^{\textrm{a}}$ Demir et al [11]

$^{{\textbf{b}}}$ Saha et al [50]

$^{{\textbf{c}}}$ Wang et al [51]

$$^{\textrm{d}}$$ Puchalski [52]

$$^{\textrm{e}}$$ S. Bashkin [1] $$+$$ Johansson [2]

**Fig. 1:**
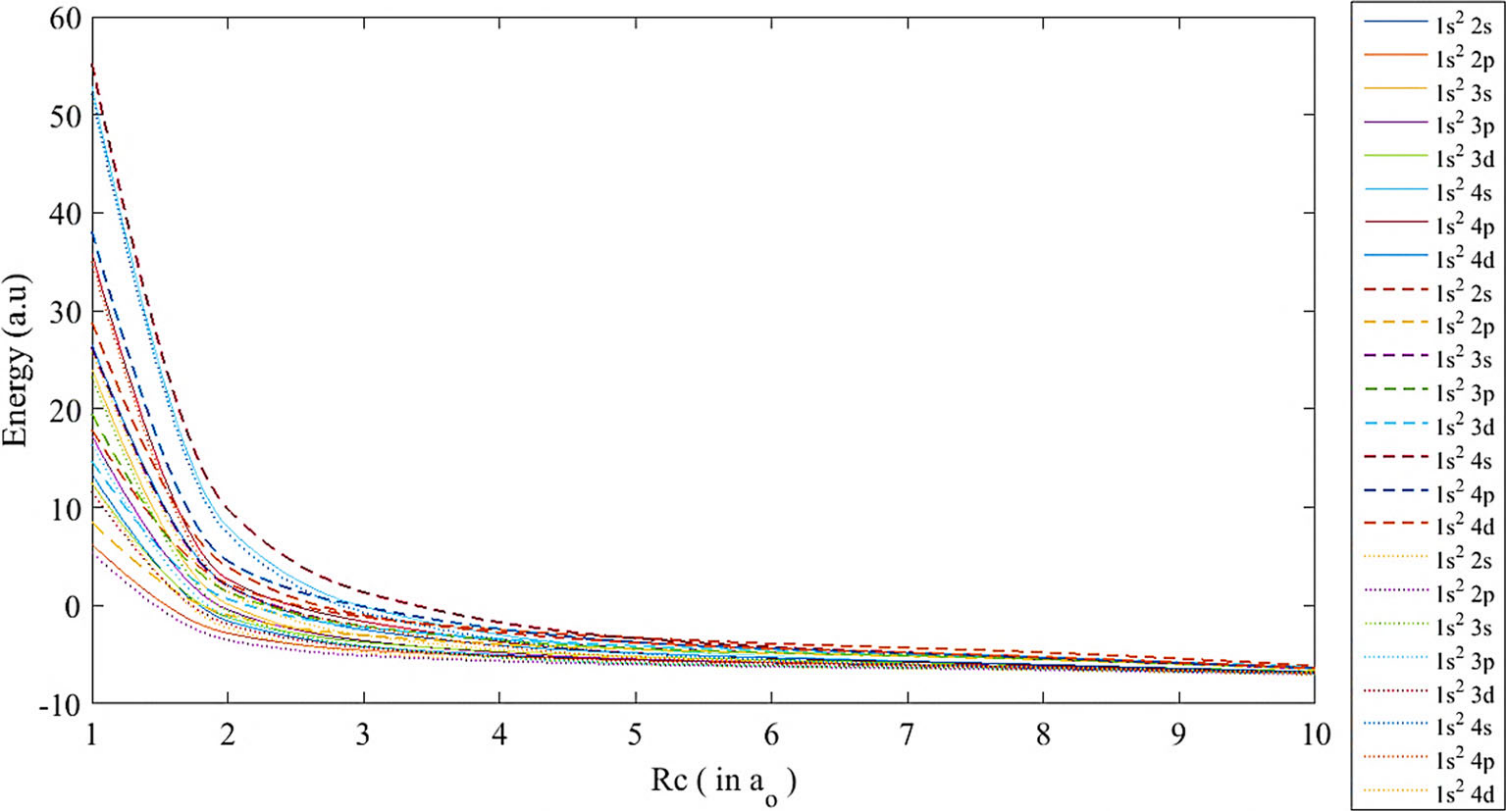
The ground and low-lying excited states of lithium atom using the SCP (-), ECSCP (—), and Hulthén (...) models at $\mu \,=$ 0.1 and various values of *Rc*

**Fig. 2:**
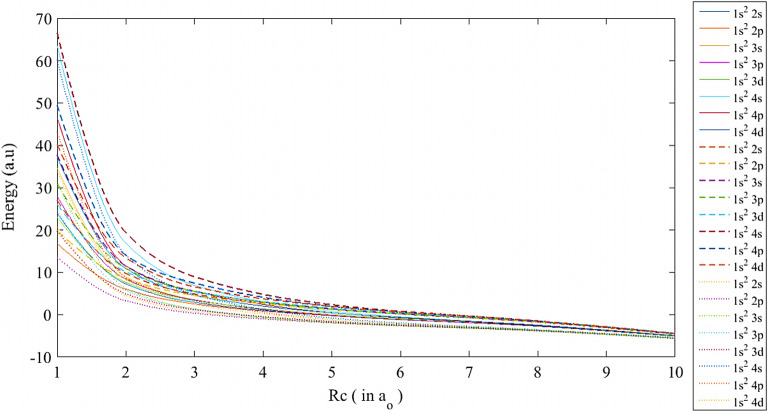
The ground and low-lying excited states of lithium atom using SCP (-), ECSCP (—), and Hulthén (...) models at $\mu \,=$ 0.5 and various values of *Rc*

**Fig. 3:**
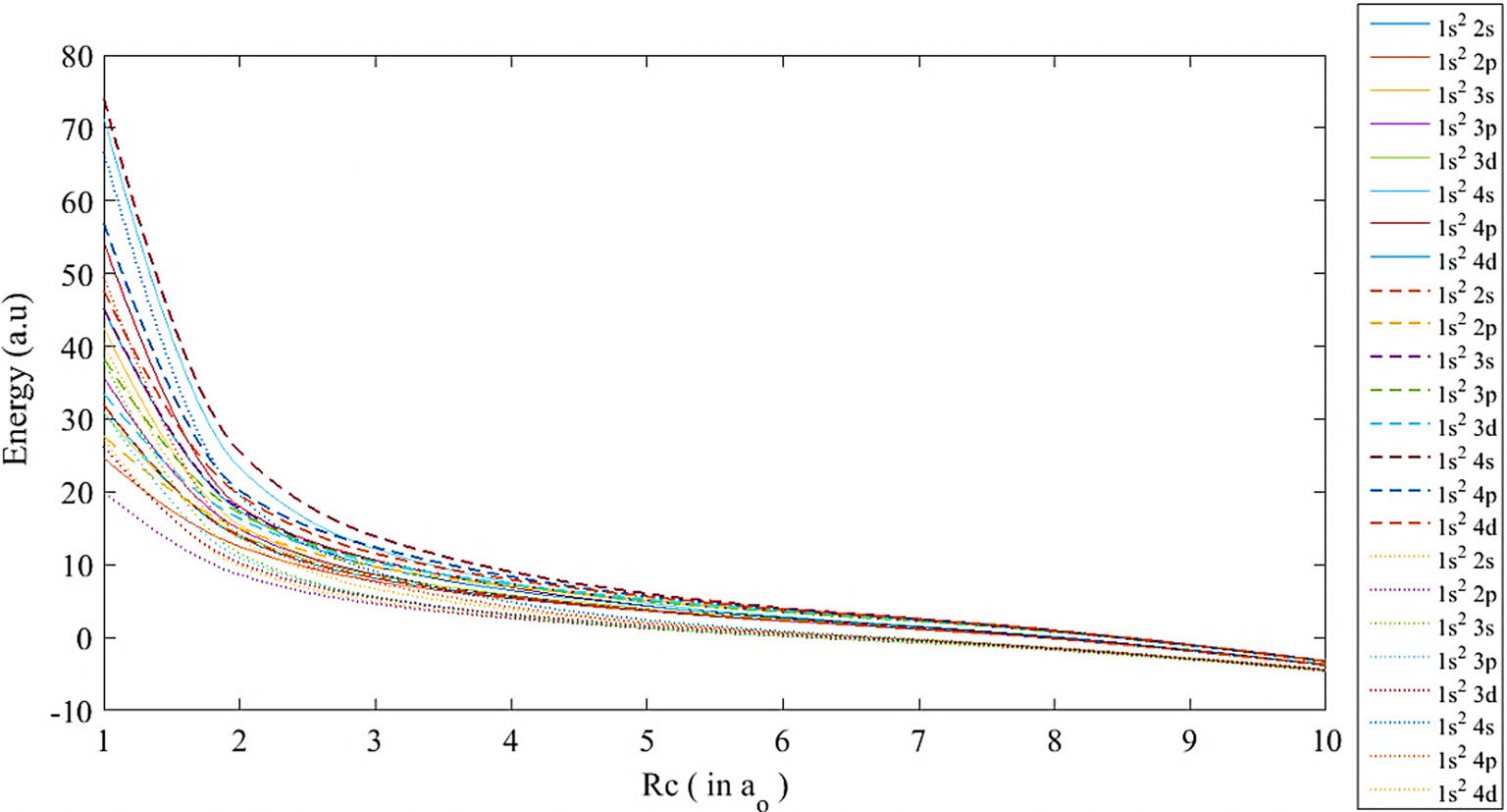
The ground and low-lying excited states of lithium atom using the SCP (-), ECSCP (—), and Hulthén (...) models at $\mu \,=$ 1 and various values of *Rc*

Considering our first case, in the presence of the compression impact only, the calculations were performed for the ground and low-lying excited doublet states of the lithium atom using the Hamiltonian of Eq. (7) with the condition of Eq. (16). Besides, the used trial wave function is Eq. (17), including Eq. (21a) as a spin function for $1\,s^{{2}}$ *ns * and $1\,s^{{2}}$ *nd* states, while Eq. (21b) is for $1 s^{{2}}$*np* states. For the other case, the calculations were made for the compressed plasma models by the impenetrable spherical box using the Hamiltonian of Eq. (15), including Eqs. (12), (13), and (14). The trial wave functions used are given by Eq. (17) for the SCP and Hulthén models, whereas Eq. (18) is for the ECSCP model. Also, the spin wave function is Eq. (21a) for the $1 s^{{2}}$ *ns* and $1 s^{{2}}$ *nd* states, while Eq. (21b) is for the $1 s^{{2}}$*np* states. All the obtained results in the presence of the plasma models are presented in Table 1, whereas Table 2 demonstrates only the system in the free and the compression state compared to the other previous results [11, 52–54] at different confinement strengths.

At $\mu =0$ and for a strong compression at small values of *Rc*, the energy of the entire system increases monotonically and the kinetic energy becomes predominant associating with the increase in correlation energy (electron–electron repulsion), making a gradual decrease in Coulomb potential, whereas for large values of *Rc* the compression effect is not noticeable and the energy is perturbed slowly, where the system is still attractive owing to the Coulomb potential. Due to the quantum mechanical uncertainty principle, the confinement of atoms within a shrinking spherical box causes a rise in electron momentum, resulting in a buildup of kinetic energy that cannot be counteracted by the rise in Coulombic attractive energy.

Conversely, at $Rc=\infty$, the total energy of bound systems is governed by the plasma conditions, whereby it is expected that the energies of bound states are less than zero since the interaction between the nucleus and the electron still exists and the corresponding potential is still attractive, at small values of $\mu$. Then the impact of the pure plasma environment results in increasing the total energy gradually as $\mu$ gets larger and makes the bound systems reach the ionization limit rapidly, which ultimately leads to an unstable system (an unbound system). Typically, the system’s instability is primarily due to the rise in the screening parameter, leading to a continuous decrease in the pure Coulomb and correlation potentials because of the increasing electron-nucleus and inter-electronic distances. Meanwhile, the system’s kinetic energy increases and ultimately takes control of the entire system.

In case of the presence of both impacts, and for a given large value $Rc,\,$ the Coulomb and correlation energies experience a slight decrease with increasing $\mu$, demonstrating the influence of the screening potential on the nuclear charge. However, reducing the box radii results in the atom being fully compressed, leading to an elevation in both kinetic and correlation terms, in addition to the gradual reduction in the attractive Coulomb potential due to the corresponding increase in the distance between the electrons and the nucleus.

In addition, the energy eigenvalues using the three plasma models were plotted versus $Rc\,$ at different plasma screening strengths, as shown in Figs. 1, 2 and 3. At $\mu = 0.1$, as in Fig. 1, the energy levels are close to each other due to the weak impact of the plasma potential on the lithium atom at 5 $\le Rc \quad \le 10$, whereby the energy eigenvalues corresponding to each state of the same energy level get closer by increasing the atomic distances and maintaining the attractive Coulomb potential. Then, the divergence between the models starts at $Rc< 5$ and reaches the maximum energy gap at $Rc=1$. As the plasma screening parameter is raised, the divergence among all energy levels initiates at $Rc \quad \le$ 8 for $\mu \quad = 0.5$ and $\mu \quad = 1$, subsequently expanding at $Rc\,\le 6$ and $Rc\,\le 5$, as illustrated in Figs. 2 and 3, respectively. As the box radius is reduced, leading to a high-pressure state for the atom, the system becomes unstable, resulting in a complete avalanche effect for the pure Coulomb potential and a rapid increase in repulsive energy. The reasons for the shifts of the ECSCP model in the energy levels to higher positions than the other models are due to the increasing plasma density impact on the lithium atom (i.e., collisional plasma regime), as well as the increasing in the entire system kinetic energy. Additionally, there exist crossovers among the excited states of the lithium atom transitioning from $1s^{{2}}2s$ state in ECSCP to $1s^{{2}}4p$ state in SCP at $Rc\cong$ 1.8, 1.6, and 1.85, as shown in Figs. 1, 2, and 3, respectively. In Fig. 2, the crossovers include transitions from $1s^{{2}}3s$ state in SCP to $1s^{{2}}3p$ state in ECSCP at $Rc\cong$ 1.55, along with transitions from $1s^{{2}}2 s$ state in the Hulthén potential to $1s^{{2}}2 p$ state in ECSCP at $Rc\cong$ 1.58. This indicates that the incorporation of the confined model (ECSCP) demonstrated significant outcomes in comparison to the alternative plasma model by enhancing the plasma strength $\mu$.

## Conclusions

The investigation presented here makes a valuable contribution to our understanding of the behavior of compressed ground and low-lying excited doublet states of the lithium atom embedded in three various plasma potentials of different strengths. All calculations were carried out using the Metropolis algorithm within the computational variational Monte Carlo (VMC) method, yielding results with low standard deviation. In order to achieve these objectives, we employed appropriate trial wave functions, which were based on hydrogenic wave functions multiplied by the Jastrow correlation and the spin functions, as well as the cut-off factor regarding the confinement strengths and effective-plasma factor for the ECSCP model. The results obtained provided insight into the characteristics of the ground and excited states of the lithium atom when embedded in each model, particularly for the ECSCP model by decreasing the spherical box radii, which exerted a stronger plasma screening and confining effects on all states compared to the SCP and Hulthén potential models. Moreover, at large values of box radii, it increased the entire system’s kinetic energy in addition to decreasing the pure Coulomb and correlation potentials more rapidly than other models. Also, the energy values of the three models were plotted as functions of the spherical box radius at various plasma strengths to show graphically the impact of pressure on the atom by decreasing the radius value, which led to increase of the kinetic and correlation energies of all systems. The results imply that the energy sequence $E_{Hu\,}< E_{SCP\,}{< }E_{ECSCP}$ is substantiated through numerical verification utilizing the VMC approach at certain values of $\mu$and *Rc*for the lithium atom.

## Data Availability

The authors declare no competing interests.

## References

[1] S. Bashkin, J.O. Stoner, *Atomic energy levels and gotrian diagrams*, 1st edn. (North-Holland, Amsterdam, 1975)

[2] I. Johansson, Ark. Fys. **15**, 169 (1959)

[3] C. Laughlin, G.A. Victor, Adv. At. Mol. Phys. **25**, 163 (1988)

[4] S. Der-Ruenn, Chin. J. Phys. **27**, 2 (1989)

[5] T. Sako, G.H. Diercksen, J. Phys. B: At. Mol. Opt. Phys. **36**, 1433–1457 (2003)

[6] A.V. Turbiner, J.C. Vieyra, H.O. Pilón, Ann. Phys. **409**, 167908 (2019)

[7] A. Borovik, A. Kupliauskienė, J. Phys, Conf. Ser. **488**, 042004 (2014)

[8] K.D. Sen, J. Chem. Phy. **122**, 194324 (2005) 10.1063/1.1901584 16161590

[9] S.B. Doma, F.N. El-Gammal, M.A. Salem, Eur. Phys. J. D **75**, 1232021 (2021)

[10] S.H. Patil, Y.P. Varshni, Can. J. Phys. **82**, 647–659 (2004)

[11] C. Demir, Y. Yakar, B. Çakır, A. Özmen, J. Lumin. **251**, 119185 (2022)

[12] A. Özmen, B. Çakır, C. Demir, Y. Yakar, Phys. B **656**, 414775 (2023)

[13] C.Y. Lin, Y.K. Ho, J. Phys. B **45**, 145001 (2012)

[14] S. Paul, Y.K. Ho, Phys. Rev. A **78**, 042711 (2008)

[15] S. Sahoo, Y.K. Ho, Phys. Plasmas **13**, 063301 (2006)

[16] M.S. Murillo, J.C. Weisheit, Phys. Rep. **302**, 1–65 (1998)

[17] M. Bonitz et al., J. Phys. A: Math. Gen. **36**, 5921 (2003)

[18] N. Das, B. Das, A. Ghoshal, Int. J. Quantum Chem. **124**, 1 (2023)

[19] N. Das, A. Ghoshal, Phys. Plasmas **31**, 053513 (2024)

[20] G. Manfredi, Fields Inst. Commun. **46**, 263 (2005)

[21] P.K. Shukla, B. Eliasson, Phys. Lett. A **372**, 2897 (2008)

[22] S.C. Na, Y.D. Jung, Phys. Lett. A **372**, 5605 (2008)

[23] S.C. Na, Y.D. Jung, Phys. Scr. **78**, 035502 (2008)

[24] S. Dutta, J.K. Saha, R. Chandra, T.K. Mukherjee, Phys. Plasmas **23**, 042107 (2016)

[25] S.B. Doma, H.S. El-Gendy, M.A. Abdel-Khalek, M.M. Hejazi, Indian J. Phys. **95**, 2847–2853 (2020)

[26] H.W. Li, S. Kar, Phys. Plasmas **19**, 073303 (2012)

[27] L.B. Zhao, Y.K. Ho, Phys. Plasmas **11**, 1695 (2004)

[28] Y.Y. Qi, Y. Wu, J.G. Wang, Phys. Plasmas **16**, 033507 (2009)

[29] N. Masanta, A. Ghoshal, Chin. J. Phys. **71**, 273–285 (2021)

[30] A. Ghoshal, Y. Kam Ho, Phys. Rev. A **95**, 052502 (2017)

[31] A. Ghoshal, Y. Kam, J Fiz Malays. **39**, 20001–20012 (2018)

[32] N. Masanta, A. Ghoshal, Few-Body Syst. **62**, 95 (2021)

[33] K. Shuai, H. Juan, X. Ning, C. Chang-Yong, Commun. Theor. Phys. **62**, 881–887 (2014)

[34] S.B. Doma, M.A. Salem, F.N. El-Gammal, Int. J. Quantum Chem. **124**, e27255 (2024)

[35] S.B. Doma, G.D. Roston, M.F. Ahmed, J. Phys. Soc. Jpn. **93**, 034301 (2024)

[36] W.A. Lester, B.L. Hammond, Ann. Rev. Phys. Chem. **41**, 283–311 (1990)

[37] Z. Shao, Y. Tang, Physica. B. Condens. **404**, 217–222 (2009)

[38] S.C. Pieper, Nucl. Phys. A **751**, 516c–532c (2005)

[39] S.B. Doma, G.D. Roston, M.F. Ahmed, K.D. Sen, Acta Phys. Pol., A **144**, 63–68 (2023)

[40] N. Metropolis, A.W. Rosenbluth, M.R. Rosenbluth, A.H. Teller, E. Teller, J. Chem. Phys. **21**, 1087–1092 (1953)

[41] Y.B. Kumar, Nat. Astron. **4**, 1059–1063 (2020)

[42] W.M.C. Foulkes et al., Rev. Mod. Phys. **73**, 1 (2001)

[43] M.B. Ruiz, Int. J. Quantum Chem. **101**, 246–260 (2005)

[44] S. Ichimaru, H. Iyetomi, S. Tanaka, Phys. Rep. **149**, 91–205 (1987)

[45] A. Ghoshal, Y.K. Ho, Mod. Phys. Lett. B **25**, 1619–1629 (2011)

[46] L. Hulthén, Ark. Met. Astron. Fys. A **28**, 5 (1942)

[47] N.L. Guevara, F.E. Harris, A.V. Turbiner, Int. J. Quantum Chem. **109**, 3036–3040 (2009)

[48] B. Supriadi, A. Harijanto, M. Maulana, Z.R. Ridlo, W.D. Wisesa, A. Nurdiniaya, J. Phys, Conf. Ser. **1211**, 012052 (2019)

[49] W. Greiner, *Quantum Mechanics: An Introduction*, 4th edn. (Springer-Verlag, Berlin Heidelberg, Germany, 2001), p.221

[50] S.B. Doma, G.D. Roston, M.F. Ahmed, Rom. Rep. Phys. **76**, 204 (2024)

[51] C. Filippi, C.J. Umrigar, J. Chem. Phys. **105**, 213 (1996)

[52] J.K. Saha, S. Bhattacharyya, S.F. Ahmed, Int. J. Quantum Chem. **121**, e26570 (2021)

[53] L.M. Wang, Z.-C. Yan, H.X. Qiao, G.W.F. Drake, Phys. Rev. A **85**, 052513 (2012)

[54] M. Puchalski, K. Pachucki, Phys. Rev. A **73**, 022503 (2006)

